# Validation of an automated colony counting system for group A Streptococcus

**DOI:** 10.1186/s13104-016-1875-z

**Published:** 2016-02-08

**Authors:** H. R. Frost, S. K. Tsoi, C. A. Baker, D. Laho, M. L. Sanderson-Smith, A. C. Steer, P. R. Smeesters

**Affiliations:** Group A Streptococcus Research Group, Murdoch Childrens Research Institute, Flemington Road, Parkville, Melbourne, VIC 3052 Australia; Laboratoire de Bactériologie Moléculaire, Université Libre de Bruxelles, Brussels, Belgium; Illawarra Health and Medical Research Institute and School of Biological Sciences, University of Wollongong, Wollongong, Australia; Centre for International Child Health, University of Melbourne, Melbourne, Australia; Department of General Medicine, Royal Children’s Hospital Melbourne, Melbourne, Australia

**Keywords:** Automated, Colony counting, Group A Streptococcus, TTC

## Abstract

**Background:**

The practice of counting bacterial colony forming units on agar plates has long been used as a method to estimate the concentration of live bacteria in culture. However, due to the laborious and potentially error prone nature of this measurement technique, an alternative method is desirable. Recent technologic advancements have facilitated the development of automated colony counting systems, which reduce errors introduced during the manual counting process and recording of information. An additional benefit is the significant reduction in time taken to analyse colony counting data. Whilst automated counting procedures have been validated for a number of microorganisms, the process has not been successful for all bacteria due to the requirement for a relatively high contrast between bacterial colonies and growth medium. The purpose of this study was to validate an automated counting system for use with group A Streptococcus (GAS).

**Results:**

Methods: Twenty-one different GAS strains, representative of major *emm*-types, were selected for assessment. In order to introduce the required contrast for automated counting, 2,3,5-triphenyl-2H-tetrazolium chloride (TTC) dye was added to Todd–Hewitt broth with yeast extract (THY) agar. Growth on THY agar with TTC was compared with growth on blood agar and THY agar to ensure the dye was not detrimental to bacterial growth. Automated colony counts using a ProtoCOL 3 instrument were compared with manual counting to confirm accuracy over the stages of the growth cycle (latent, mid-log and stationary phases) and in a number of different assays. The average percentage differences between plating and counting methods were analysed using the Bland–Altman method.

**Conclusions:**

Results: A percentage difference of ±10 % was determined as the cut-off for a critical difference between plating and counting methods. All strains measured had an average difference of less than 10 % when plated on THY agar with TTC. This consistency was also observed over all phases of the growth cycle and when plated in blood following bactericidal assays. Agreement between these methods suggest the use of an automated colony counting technique for GAS will significantly reduce time spent counting bacteria to enable a more efficient and accurate measurement of bacteria concentration in culture.

**Electronic supplementary material:**

The online version of this article (doi:10.1186/s13104-016-1875-z) contains supplementary material, which is available to authorized users.

## Findings

### Background

The practice of counting bacterial colony forming units (CFU) on agar plates has long been used as a method to estimate the concentration of live bacteria in a culture. However, due to the laborious and potentially error prone nature of this measurement technique, an alternative method is desirable. Recent technologic advancements have facilitated the development of automated colony counting systems, which remove the potential for error introduced both during the manual counting process and in the recording of this information [1]. Automated counting procedures have been validated for a number of microorganisms, including *Pseudomonas aeruginosa* and *Streptococcus pneumoniae*, grown on various agar media [1, 2]. However automated counting has not been successful for all bacteria due to the requirement for a relatively high contrast between the bacteria colonies and growth medium. Group A *Streptococcus* (GAS) is a Gram-positive, beta-haemolytic bacterium that is an important cause of infectious disease morbidity and mortality worldwide [3]. The characteristic beta-hemolysis of GAS on blood agar plate makes automatic colony counting more challenging because of the resulting poor contrast between the colony and the growth medium.

2,3,5-Triphenyltetrazolium chloride (TTC) is a redox indicator that produces water insoluble red formazan crystals when reduced [4]. It has been used extensively for several decades as an indicator of tissue ischaemia [5, 6], and more recently to detect the presence of live micro-organisms based on metabolic activity of intracellular enzymes [4]. Accumulation of TTC within live bacteria provides high contrast between bacterial colonies and several solid agar media. TTC has been successfully used for colony staining for automated colony counting in gram positive organisms including *S. pneumoniae* and group B Streptococcus [2, 7]. One study reported toxicity of TTC to *Listeria monocytogenes* but whether this phenomenon occurs in GAS is unknown [8].

A number of vaccine candidates against GAS are currently under investigation in clinical trials and in pre-clinical studies [9]. Bactericidal assays are essential for the evaluation of efficacy in these trials, which require the enumeration of many bacterial cultures and as such are very time consuming. In order to increase the throughput and reproducibility of immunoassays involved in GAS research, we developed a method for automated counting of GAS CFU on Todd Hewitt broth agar plates with TTC dye. Our study aimed to validate this method using a collection of representative GAS isolates in different experimental procedures and conditions.

### Methods

#### Bacteria strains and culture

Twenty-one GAS strains belonging to 21 different *emm*-types were selected representing the 13 most frequent *emm*-clusters globally ([10]; Smeesters Personal Communication; Table 1). Glycerol stocks of GAS were streaked on Colombia Horse Blood Agar (HBA; Thermo-Fisher Scientific, Scoresby) plates and incubated overnight at 37 °C with 5 % CO_2_ to obtain single colonies [11]. Individual colonies were used to inoculate Todd Hewitt broth with 1 % yeast extract (THY). Liquid cultures for all strains were incubated at 37 °C with agitation at 100 rpm. A tenfold serial dilution of each culture was made after incubation to mid-log phase and dilutions of 1:100 to 1:10^6^ were plated in triplicate. Using a multichannel pipette, 5 µL of dilutions were dripped onto the plates and allowed to spread vertically (Fig. 3). Plates were dried for 10 min and incubated overnight at 37 °C with 5 % CO_2_.

**Table 1:** GAS Strains analysed on different media in various assays

*emm*-type	*emm*-cluster	Mid-log phase HBA	Mid-log phase THY-agar	Growth curve	Bactericidal assay
1	A-C3	+	+	−	−
2	E4	+	+	+	+
3	A-C5	+	+	−	−
4	E1	+	+	+	−
11	E6	+	+	−	+
12	A-C4	−	−	+	+
17	Clade Y	+	−	−	−
25	E3	−	−	−	+
33	D4	+	−	+	−
36	D1	+	−	−	−
46	A-C1	+	−	−	−
58	E3	+	−	−	+
60	E1	+	−	−	−
65	E6	+	−	−	+
66	E2	+	−	−	−
75	E6	+	−	−	+
81	E6	−	−	−	+
89	E4	−	−	−	+
100	D2	+	−	−	−
103	E3	−	−	−	+
230	D4	+	−	−	−

#### Plating bacterial cultures

**Fig. 1:**
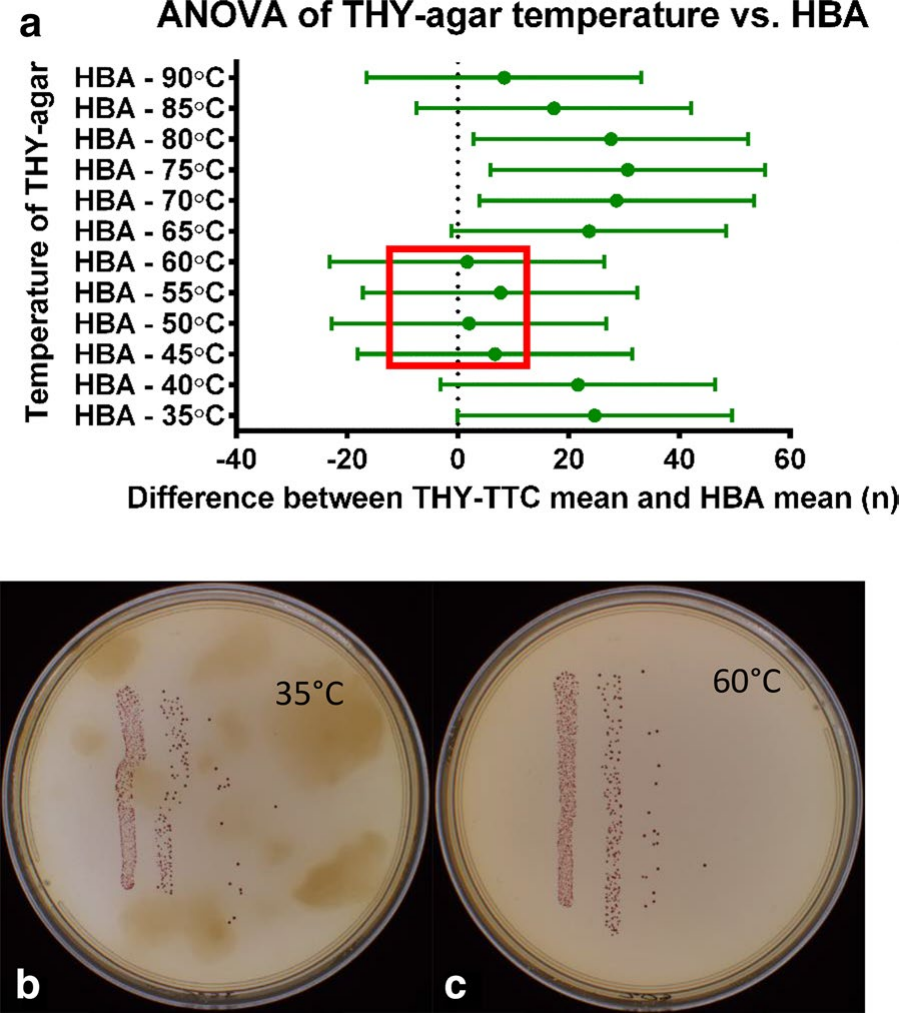
Analysis of the effect of different temperature media when TTC is added. **a** Analysis of variance of colony counts of an M25 culture serially diluted and plated in triplicate on agar prepared with addition of TTC at a range of temperatures. The average colony count from the 10^−3^ dilution on plates prepared at each temperature were compared with the average colony count of the same culture plated on HBA. The *whiskers* represent 95 % confidence intervals around the means. **b** Example of a plate poured when TTC was added at 35 °C and **c** at 60 °C and used to plate GAS cultures

HBA plates were sourced commercially and THY-agar plates were made by addition of 1 % agar to THY prior to autoclaving. TTC (Sigma Aldrich, Sydney) was diluted in PBS and filter sterilised through a 0.22 µm syringe-driven filter before being added at a final concentration of 0.04 mg/mL to autoclaved THY-agar to make THY-TTC plates. As TTC is heat-sensitive, addition of TTC to agar prior to adequate cooling results in the reduction of dye to the coloured form 1,3,5-triphenylformazan (TPF) which in turns lead to insufficient coloration of live bacteria and lower contrast between colonies and agar. However with excessive cooling the agar solidifies and the media is unable to be poured into plates (Fig. 1). The optimum range of agar temperatures for the addition of TTC and pouring plates was examined by cooling the agar to between 35 and 90 °C prior to addition of TTC. A GAS culture was diluted and plated on plates prepared from all temperatures measured and HBA. The time for which the THY-TTC plates remain usable was examined over 4 weeks. The older plates had been stored at 4 °C and were incubated at room temperature for 3 h before use and the fresh plates were left at room temperature following pouring.

#### Counting colonies

Bacterial colonies plated on THY-agar and HBA were counted manually by one person, by photographing the plates and counting with ImageJ software (http://imagej.nih.gov/ij/) which marks each colony and counts the total. Where visible, touching colonies were split and counted as multiple colonies. Bacterial colonies plated on THY-TTC were counted using the Protocol 3 instrument with the automatic colony counting functionality according to manufacturer’s recommendations [12]. Briefly, batches were configured to the plate diameter and for a 5 µL sample and exposure was set at 12 ms. Colour configuration was repeated for each batch based on an exemplar plate, small particles were included and the colonies that appeared to be touching were split where applicable. Frames and zones were modified as necessary to fit all colonies from each drip and were carefully examined to ensure all colonies and no debris or artefacts were included in the count. For a full protocol on plate preparation and using the automated colony counting system please refer to Additional file 1. Time needed to perform manual and automatic counting was recorded.

#### Experiments tested

##### Growth curve

All stages of the bacterial growth cycle were tested by automatic counting to ensure reliability of the methodology at the different phases. GAS grown overnight on HBA plates were used to inoculate THY media and incubated for 2 h at 37 °C with agitation at 100 rpm. After 2 h, samples of the cultures were diluted tenfold serially and plated on HBA and THY-TTC agar, and further samples were used to measure the optical density at 600 nm (OD_600_). The cultures were returned to the incubator and the above repeated every hour until a plateau was reached according to the OD_600_ of the cultures. Plates were incubated overnight at 37 °C with 5 % CO_2_.

##### Mid-log phase

Previous studies had identified a potential toxic effect of TTC on gram-positive bacteria during mid-log phase [8]. Therefore toxicity of TTC was investigated by comparison of bacterial growth on the different agar media. Sixteen strains of GAS were grown to mid-log phase, cultures were diluted in PBS and plated in triplicate on HBA, THY-TTC or THY agar plates.

##### Bactericidal assay

In order to determine whether the presence of TTC has any detrimental effect on the estimation of bactericidal activity in immunoassays, a number of direct bactericidal assays (DBA) were undertaken on a range of strains and donors. The protocol was approved by the Ethics Committees of Erasme Hospital in Brussels, Belgium (Reference: P2015/398/B406201525683), and written informed consent was obtained from each participant. Mid-log phase GAS cultures were diluted in PBS to 1 × 10^−3^, 4 × 10^−3^ and 1.6 × 10^−4^ and used to inoculate fresh whole blood at a ratio of 1:4. Bacteria and blood cultures were incubated for 3 h at 37 °C with rotation [11]. Following incubation, cultures were diluted tenfold in PBS and plated on THY-TTC and HBA plates as above. The original diluted inoculum was also plated prior to the 3 h incubation (T_0_) and the fold-increase between the two was calculated to estimate survival of bacteria in blood.

#### Statistical analysis

Growth of bacteria on different agar media was compared using a mean-difference test and Bland–Altman plot of the percentage difference against the average [13], using GraphPad Prism (http://www.graphpad.com/). A percentage difference analysis was employed by which the difference between colony counts on THY-TTC and THY or HBA were divided by the mean of the two counts. This percentage difference was plotted on the Y axis and the mean of the two counts was plotted on the x axis. The bias is the mean of the percentage difference values and if the value is positive it reflects higher counts from the THY-TTC plates than the other method measured. The cut-off for significant percentage difference was 10 % and dilutions resulting in colony counts of under ten and over 200 were not included in analyses since plates with these extreme number of colonies do not provide accurate counting material [14, 15]. All cultures used were diluted tenfold serially in PBS and dilutions of 10^−2^–10^−6^ were plated in triplicate. Comparisons between manual and automatic counting were made between each colony count from each technical replicate plated on both agar-media.

### Results and discussion

#### THY-TTC plate preparation

We sought to determine the optimum temperature range for the preparation of THY-TTC agar plates. Analysis of the variance of the different temperatures compared with plating on HBA as a control (Fig. 1) indicates cooling and maintaining autoclaved agar between 45 and 60 °C prior to addition of TTC and pouring.

#### THY-TTC plate expiration

**Fig. 2:**
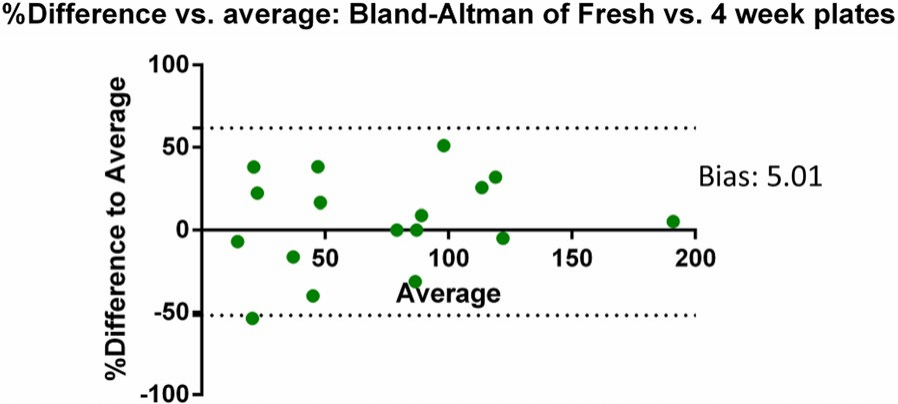
Comparison between freshly prepared THY-TTC plates and plates stored at 4 °C for 4 weeks. The analysis compares each replicate of each dilution with the sample from the same culture plated on the 4 week old plates and the fresh plates. The *Y axis* is based on the formula [100 × (4 week old plates–fresh plates)/average of the two points] and the *X axis* is the average of the two points. The bias of 5.01 indicates that plates maintain their utility for this period. The *dotted-lines* represent the 95 % limits of agreement and range from −51.79 to 61.80. For the comparison between the plates, a series of three dilutions were plated for four strains in duplicate, of which 17 counts fell between 10 and 200 colonies. *Each point* represents one dilution point of each replicate

In order to ensure the THY-TTC agar plates remain viable for a similar period to other agar plates, a comparison was made between plates prepared the day before the assay and plates prepared 4 weeks prior. Figure 2 shows that there was minimal variance between the CFU counts between fresh and 4 weeks old plates, indicating that the plates are suitable for use after storage for at least 4 weeks.

#### Growth of mid-log phase GAS on different plating media

**Fig. 3:**
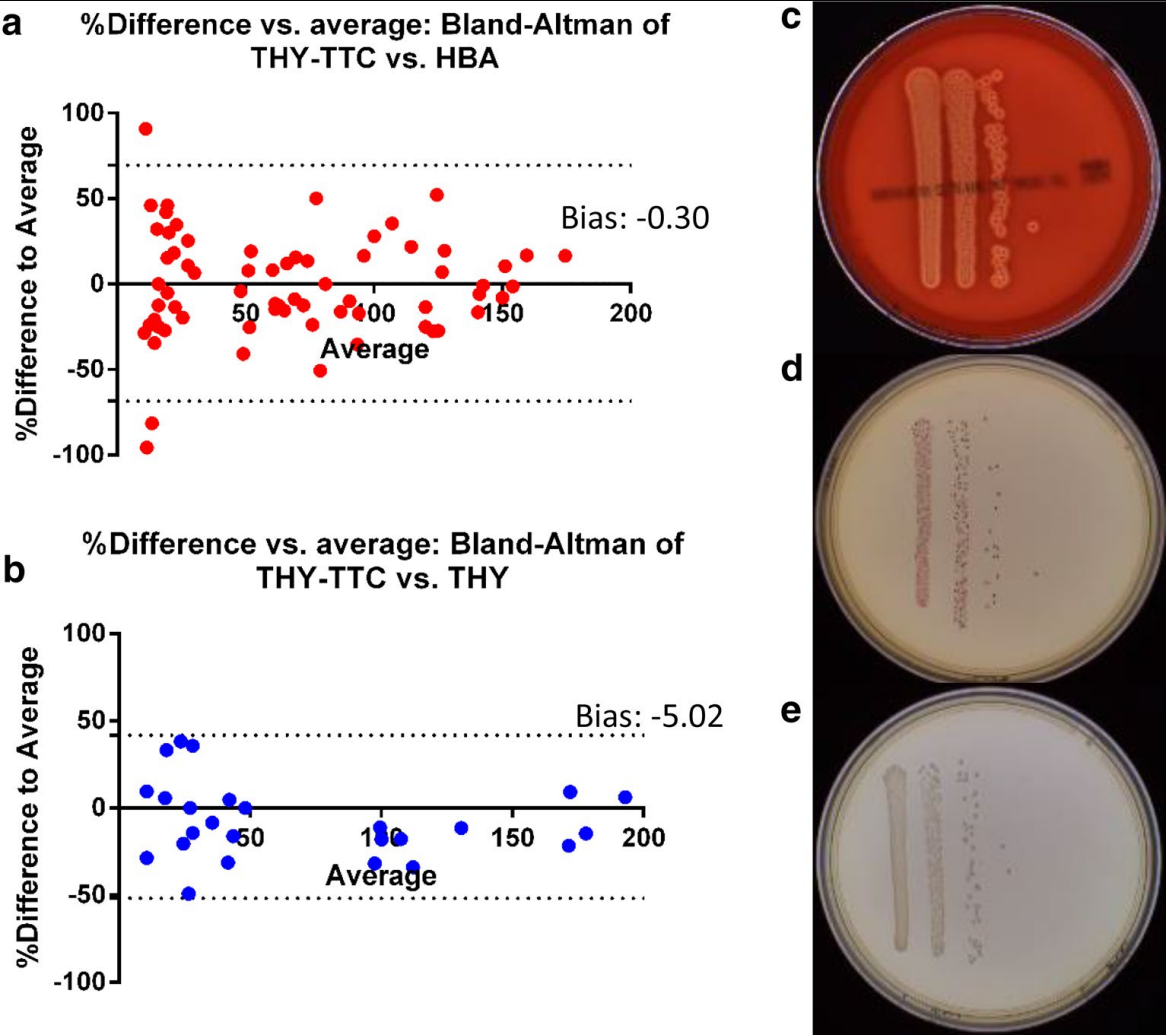
Growth of mid-log phase GAS on different plating media. Bland–Altman percentage difference of THY-agar, HBA and THY-TTC. The plots compare each replicate of each dilution with the sample from the same culture plated on THY-TTC agar and either HBA (**a**) or THY-agar (**b**). The *Y axis* is based on the formula [100 × (THY-TTC agar–HBA or THY agar)/average of the two points] and the *X axis* is the average of the two points. The biases of −0.30 and −5.02 are less than the cut-off of ±10 % which show plating on THY-TTC is sufficiently analogous to plating on either of the currently accepted methods. The *dotted-lines* represent the 95 % limits of agreement and range from −69.09 to 68.48 with HBA and −51.83 to 41.80 with THY agar. **c**–**e** show representative images of cultures grown on HBA, THY-TTC and THY-agar respectively. For the comparison with HBA, a series of five dilutions was plated for 16 strains in triplicate, of which 70 counts fell between 10 and 200 colonies. For the comparison with THY-agar, a series of five dilutions was plated for five strains in triplicate, of which 26 counts fell between 10 and 200 colonies

Strains representing the major *emm*-clusters were grown for 3–4 h to be at the mid-log phase of their respective growth cycles. Figure 3 shows the comparison between plating on THY-TTC and counting with automated colony counting and plating on HBA and manually counting colonies from 16 different strains (Table 1). GAS strains were selected to be representative of the major *emm*-clusters to ensure the broad range of M-protein variants were represented, as this protein probably plays an important role in the specific virulence of the strains [11, 16, 17]. All strains tested grew on THY-agar with TTC and were countable with the automated counting system, despite occasional morphological colony variance. Figure 3 also shows the comparison between THY-TTC and THY-agar with no dye added to examine any detrimental effect of TTC on the growth of bacteria for strains *emm*1, *emm*2, *emm*3, *emm*4 and *emm*11. As the cut-off for agreement between the methods was ±10 % these analyses support the use of THY-TTC agar plates and automated colony counting. Importantly, the automatic colony counting allows for significant reduction in the time needed to count bacteria. The automatic counting protocol requires 30–40 s per plate while the manual counting takes and average of 4 min per plate.

#### Effect on plating bacteria in blood

**Fig. 4:**
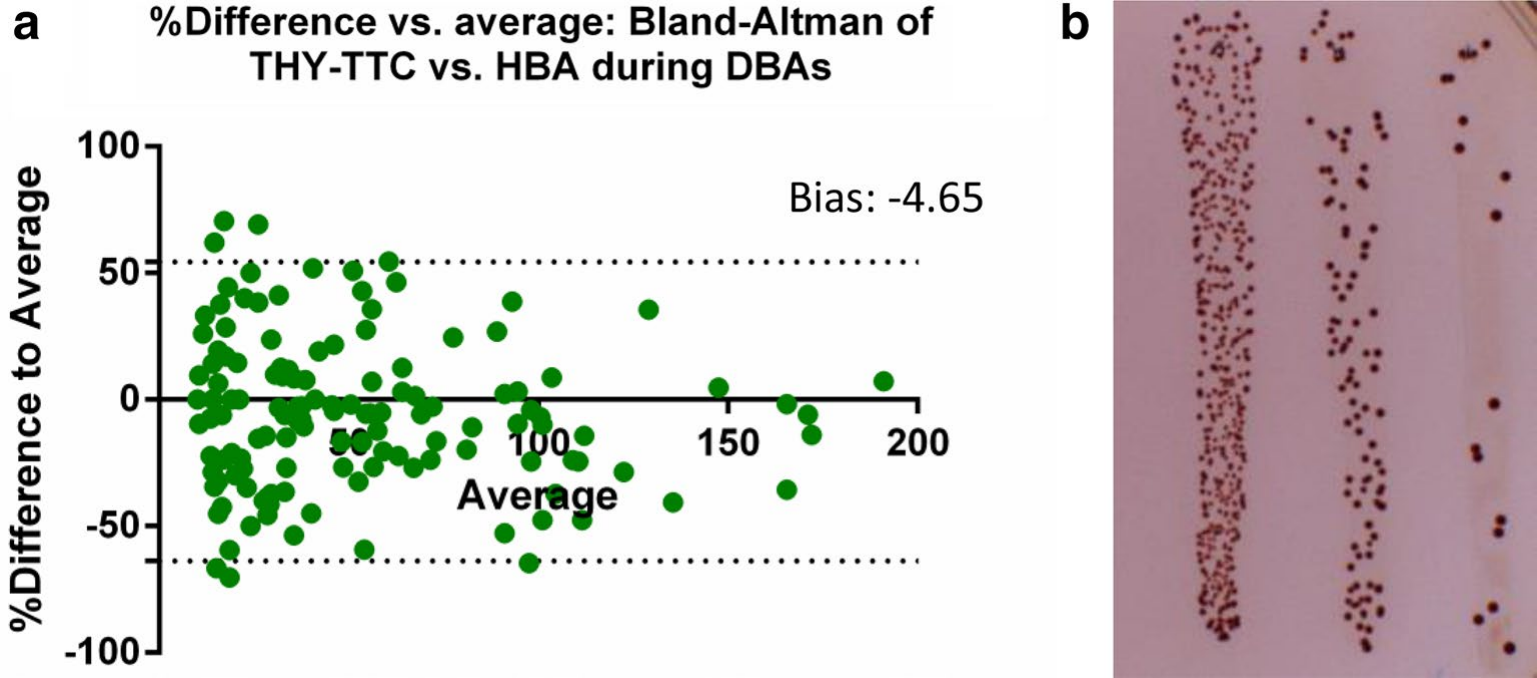
Effect on plating bacteria in blood. **a** shows the percentage difference between plating the results of a direct bactericidal assay of 10 GAS strains with three donors. The bias of −4.654 % is less than the cut-off of ±10 %. The analysis compares each replicate of each dilution with the sample from the same culture plated on HBA plates and THY-TTC plates. The *Y axis* is based on the formula [100 × (HBA THY-TTC plates–THY-TTCHBA plates)/average of the two points] and the *X axis* is the average of the two points. The bias of −4.65 indicates that the presence of blood on the agar does not significantly alter the ability of GAS to grow on both HBA and THY-TTC plates. The *dotted-lines* represent the 95 % limits of agreement and range from −63.78 to 54.47. A series of three dilutions was plated for the ten strains for each sample in triplicate, of which 36 counts fell between 10 and 200 colonies. **b** is an example of cultures plated in blood, the decreased contrast between the colonies and surrounding media does not seem to have a detrimental effect on automated counting

Bactericidal assays were performed to determine if there was a difference between the plating methods in the presence of blood. Figure 4 is a compilation of numerous DBAs, performed on ten different GAS strains (Table 1) with blood from three different donors, plated on THY-TTC and HBA. These analyses were performed to confirm the contrast remains sufficient in the presence of blood and also that there was no effect on the dye by components of blood. The observed bias of −4.65 indicates this plating and counting method is suitable for use in the bactericidal assays.

#### Efficacy in phases of growth cycle

**Fig. 5:**
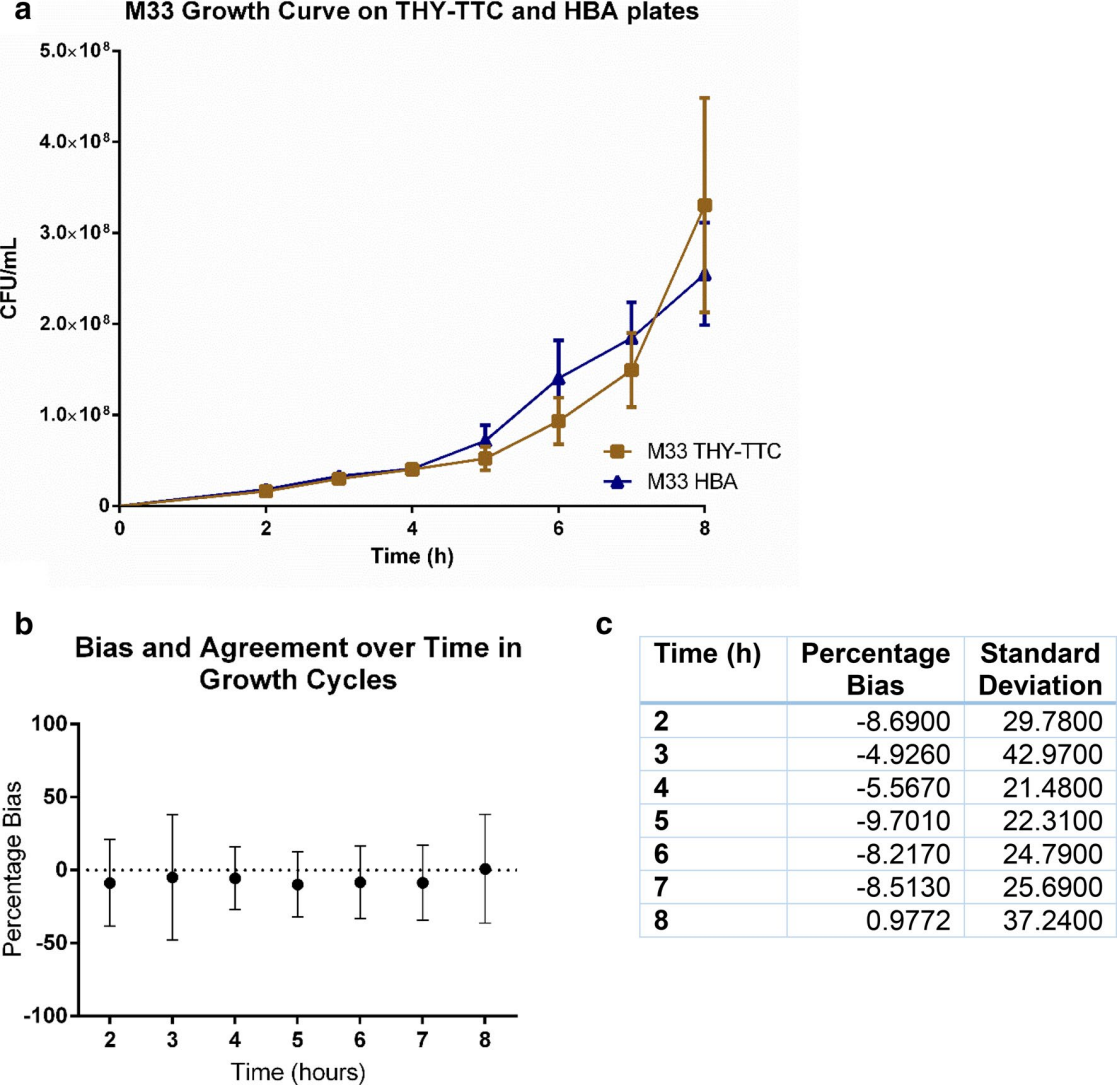
Efficacy of automated counting system over growth cycles. **a** An exemplar growth curve showing the average concentration of bacteria over the different phases of the growth curve for GAS strain M33 plated on THY-TTC and HBA. At each point the standard error of the means are overlapping. **b** Comparison of the average bias from Bland–Altman analysis at each time point of growth curves performed for four strains in triplicate. The analysis compares each replicate of each dilution with samples from the same culture plated on HBA plates and THY-TTC plates. The bias is based on the formula [100 × (THY-TTC plates–HBA plates)/average of the two counts] and is averaged for each of the 8 h of growth. As none of the biases fall outside the 10 % cut-off this indicates that plating on THY-TTC is not detrimental at any particular phase of the growth cycle. A series of three dilutions was plated for the four strains for each time point, of which 652 counts fell between 10 and 200 colonies (2 h: 90; 3 h: 104; 4 h: 111; 5 h: 103; 6 h 108; 7 h: 86; 8 h: 50). **c** The percentage biases and standard deviations

As bacteria are known to express different genes over the various stages of the growth cycle, growth curves were performed on a number of strains to determine whether the presence of TTC caused inhibition to bacterial growth at any stage. Figure 5 shows growth curves performed for four strains in triplicate (M2, M4, M12 and M33) in which the two plating methods are in agreement. Whilst TTC has been previously shown to be detrimental to the growth of gram-positive bacteria, particularly during lag phase and at high TTC concentration [8], we have not detected a substantial difference between plating methods at any stage in the life cycle. As the detrimental effect was observed with TTC concentration of 0.5 mg/mL and was reversed when the concentration was lowered to 0.01 mg/mL, the concentration utilised in our study of 0.04 mg/mL seems to be non-toxic to the bacteria.

### Conclusion

In conclusion, we have shown that the use of TTC and an automated counting system for GAS is a viable alternative for the process of counting bacterial CFU, in a number of common assays and with a broad range of *emm*-types and *emm*-clusters. A detailed protocol of our final method is provided in Additional file 1. This method will aid to avoid the introduction of errors by manually counting, in addition to being far less time consuming. The automated counting system described herein will therefore be a useful addition to GAS research protocols, and further study may allow it be used for other beta-haemolytic streptococci such as the group B Streptococcus.

#### Availability and requirements

The protocol for preparing the necessary agar plates and using the automated counting system has been provided in Additional file 1. Further information can be found on the Manufacturer’s website:

Project name: Automatic colony counting and zone measuring.

Project home page: http://www.synbiosis.com/protocol-3/.

Operating system(s): Win7 compatible.

Programming language: NA.

Other requirements: Requires connection to a stand-alone desktop or laptop computer.

License: MS Excel licence required for some functions.

Any restrictions to use by non-academics: NA.

#### Availability of supporting data

The data set supporting the results of this article is included within the article and its Additional file 1.

## Additional file

10.1186/s13104-016-1875-z Protocol GAS Automated Colony Counting on THY-TTC Agar. A working protocol for the preparation of THY-TTC agar plates, plating bacterial cultures and enumerating the resulting colonies with a Protocol-3 automated colony counting system.

## Abbreviations

CFU: colony forming unit
DBA: direct bactericidal assay
GAS: group A Streptococcus
HBA: horse blood agar
THY: Todd–Hewitt broth with yeast extract
TPF: triphenylformazan
TTC: triphenyl tetrazolium chloride
