# Supplementary material for: Validation of an automated colony counting system for group A Streptococcus
Source: BMC Res Notes. 2016 Feb 8;9:72. doi: 10.1186/s13104-016-1875-z (PMC4745170; doi:10.1186/s13104-016-1875-z)
Supplement: Supplementary file 1 — 10.1186/s13104-016-1875-z Protocol GAS Automated Colony Counting on THY-TTC Agar. A working protocol for the preparation of THY-TTC agar plates, plating bacterial cultures and enumerating the resulting colonies with a Protocol-3 automated colony counting system. [file 13104_2016_1875_MOESM1_ESM.pdf]

Protocol: GAS Automated Colony Counting on THY-TTC Agar

Plate Preparation:

1. Weigh out appropriate measures of THB, yeast extract and agar powders for THY-agar:

Todd Hewitt broth	30g/L
1% Yeast Extract	10g/L
Agar	10g/L

2. Autoclave media at 121°C for 15 minutes and allow to cool to around 55°C (no lower than 45°C and no higher than 60°C)
3. Meanwhile, dissolve TTC powder in sterile PBS (pH 7.2) to a suitable concentration e.g. 10mg/mL
4. Filter sterilise TTC through a 0.22µm syringe-driven filter unit (dissolved stock of TTC can be made in advance and stored at 4°C in a light-excluding vessel)
5. Combine TTC with cooled agar to a final concentration of 0.04mg/mL and mix by swirling gently. Pour agar into sterile petri dishes (around 20mL per 90mm plate) and allow to dry with the lid ajar for 10 minutes
6. Wrap plates with aluminium foil and store overnight at room temperature then subsequently at 4°C. Plates remain viable for at least 1 month

Plating Cultures:

1. Perform assay as required
2. Remove 100µL of bacterial culture and create a series of 10-fold dilution in sterile PBS to 10^{-6}
3. Plate 5µL drips of dilutions 10^{-2} to 10^{-6}, allow to dry and incubate plates overnight at 37°C with 5% CO_2

Automated Colony Counting:

1. Place an example plate from the batch in the plate reader
2. Select new batch and configure the plate (e.g. diameter 90mm, sample volume 5µL)
3. Select the number of rows and columns used
4. Enter names for the batch and the plate IDs
5. Select "classification" tab and "colour" classification, select "one colour" and click "next" to proceed
6. Adjust the settings to the accept the smallest size, uncheck the "reject small particles" selection, check the "split colonies" option
7. Click "next" and then "finish" to proceed
8. To adjust the frame/zone click the icon button in the top left corner and select "batch management" and "manage restrictions"
9. Choose to adjust the frame (all zones) or the zones (individual zones) to fit the colonies – include all colonies where reasonable
10. Select "measure" tab and click "test measure plate"

11. If the colonies are selected (green squares) as expected accept the new batch, otherwise zones can be readjusted and the colour reclassified. It may be necessary to manually select the colour of the background and colour of colonies.
12. Place the first plate in the colony counter, name the plate appropriately and click "test measure plate”
13. If the test count appears appropriate click “measure” to count the colonies in the zones.
    N.b. If the counting zones overlap with the edge of the plate or other defects which produce a certain amount of contrast, the counter may count excess colonies. Before you have measured the plate you can use the “exclude zone” function to remove a portion of the plate from the counter. If you have already measured the plate, select the plate ID from the list that appears below the plate display and select the “edit results” tab on the right of the screen. This tab includes functions that allow the user to add or remove individual colonies from the count or to exclude entire regions. Once you have finished click the “save changes” button and press OK.
14. After all plates in the batch have been counted, select the “results” tab on the right of the screen and then “create report” and select appropriate report type for assay.
